# Hospital Incidence and Treatment Outcomes of Patients with Aneurysms and Dissections of the Iliac Artery in Switzerland—A Secondary Analysis of Swiss DRG Statistics Data

**DOI:** 10.3390/jcm13082267

**Published:** 2024-04-14

**Authors:** Roland Bozalka, Anna-Leonie Menges, Alexander Zimmermann, Lorenz Meuli

**Affiliations:** 1 Department of Vascular Surgery, University Hospital Zurich (USZ), University of Zurich (UZH), CH-8091 Zurich, Switzerland; 2 Copenhagen Aortic Centre, Department of Vascular Surgery, Copenhagen University Hospital, 2100 Copenhagen Ø, Denmark

**Keywords:** aneurysm, iliac, artery, iliac, dissection, iliac, secondary data analysis, DRG

## Abstract

**Background/Objectives**: Aneurysms and dissections of the iliac artery (ADIAs) are significant vascular conditions often associated with aortic pathologies. Despite their importance, reports on isolated iliac artery pathologies are rare. This study aimed to investigate the epidemiology of ADIA in Switzerland including treatment incidence and hospital outcomes. **Methods**: A retrospective analysis of diagnosis-related group (DRG) statistics from 2011 to 2018 in Switzerland was conducted, identifying all cases of ADIA while excluding those with concomitant treatment of aortic pathologies. Age-standardized incidence rates and treatment outcomes were assessed, with multivariable logistic regression performed to identify factors associated with hospital mortality. **Results**: From 2011 to 2018, 1037 ADIA cases were hospitalized in Switzerland. Incidence rates for elective treatment were significantly higher in men than women, increasing in men from 1.5 to 2.4 cases per 100,000 men (*p* = 0.007), while remaining stable in women at around 0.2 cases per 100,000 women. Acute treatment incidence rates were lower but still higher in men, at 0.9 cases per 100,000 men and 0.2 cases per 100,000 women. Crude hospital mortality rates were lower for endovascular repair than open surgical repair in both elective (0.8% vs. 3.1%, *p* = 0.023) and emergency treatment (6.7% vs. 18.4%, *p* = 0.045). Multivariable analysis showed that endovascular repair was associated with significantly reduced hospital mortality compared to open repair (OR 0.27, 95%-CI: 0.10 to 0.66, *p* = 0.006). **Conclusions**: This nationwide study of iliac artery pathologies shows that the treatment incidence was about 10 times higher in men than in women for elective procedures, but only about five times higher for emergency treatment. Endovascular procedures were associated with significantly lower hospital mortality than open procedures, while hospital mortality rates were comparable for men and women.

## 1. Introduction

An iliac artery aneurysm (IAA) is the dilation of the common iliac artery (CIA), the internal iliac artery (IIA), or the external iliac artery (EIA) by more than 1.5 times the normal diameter [1]. Different classification systems exist for IAA. Historical Swedish autopsy data on aortic and iliac aneurysms from the 1970s to 1980s show that about 17% of all abdominal aortic aneurysms (AAA) are accompanied by IAA [2]. These pathologies are generally referred to as aorto-iliac aneurysms. In clinical practice, the treatment of AAA often requires simultaneous treatment of the CIA, as these vessels are frequently ectatic or aneurysmal and do not allow either endovascular sealing or durable surgical suturing. Isolated IAAs were found in only 0.7% of all patients with aorto-iliac aneurysms. Thus, the treatment of isolated IAA pathologies remains rare and there is no solid and up-to-date data on the epidemiology of isolated IAAs. For isolated IAAs, Reber’s classification according to anatomical extent has become established [3]. It comprises four types, with type I comprising isolated CIA aneurysms, type II isolated IIA aneurysms, type III a combination of CIA and IIA aneurysms, and type IV aneurysms in which all iliac vessels are dilated. The CIA is most affected, whereas the EIA is rarely affected by aneurysmal degeneration, possibly due to its different embryological origin [3]. 

The pathogenesis of IAA is similar to that of AAA and generally includes atherosclerotic degeneration of the medial wall [1]. Other etiologies include post-dissection aneurysms, infected native aneurysms, traumatic aneurysms, or pseudoaneurysms [1]. As with AAA, most patients with isolated degenerative IAA are male (90%) and usually over 70 years old at diagnosis [1].

IAA are generally asymptomatic but might cause symptoms due to compression of surrounding structures such as the ureter, sacral plexus, or iliac vein [4,5]. As with aortic aneurysms, the risk of a life-threatening rupture increases with increasing diameter [1,6,7]. However, the natural history of IAA is less well established than for AAA. The diameter threshold where elective IAA treatment should be considered has recently been increased to ≥4.0 cm (class of recommendation IIa, level of evidence C) by the European Society of Vascular Surgery (ESVS) [1]. In contrast, limited data exist on isolated dissections of the iliac arteries. Most often, case reports describe iatrogenic dissections. Reports on spontaneous dissections of the iliac arteries often involve underlying pathologies such as fibromuscular dysplasia or connective tissue diseases [8,9]. As for aortic dissections, emergent surgical treatment is generally indicated when there is evidence of rupture, or malperfusion (i.e., limb ischemia). For chronic dissections treatment is generally recommended if there is a concomitant IAA ≥ 4.0 cm [1], or can be considered in patients with clinically relevant malperfusion of the limb [10]. Treatment options encompass both endovascular and open procedures. While open therapy was the standard until the 1990s, the rapid advancement of endovascular techniques has shifted the trend towards endovascular approaches. As with AAA, endovascular procedures are associated with lower complication rates and shorter hospital stays [11]. The current ESVS guidelines recommend that the choice of surgical technique for IAA should be based on individual patient and lesion characteristics (Class IIa, Level B) [1].

This study aimed to describe the epidemiology of isolated IAA and iliac artery dissections through a secondary data analysis of Diagnosis Related Group (DRG) statistics in Switzerland. 

## 2. Materials and Methods

### 2.1. Data Source

This is a secondary data analysis of case-related hospital discharge data from the Swiss Federal Statistical Office (SFSO). Every medical institution in Switzerland, including hospitals, birthing centres, and medical specialty institutions, is bound to report all hospitalizations to the SFSO annually. The SFSO collects baseline characteristics like age, sex, and insurance class, as well as a primary diagnosis and up to 49 secondary diagnoses. Diagnoses are recorded using the 10th revision of the International Classification of Diseases (ICD-10). Further, general information like the type of admission (planned or emergent), the total length of stay in days, length of stay in the intensive care unit (ICU) in hours, and discharge information, including hospital mortality, are recorded. Finally, up to 100 procedure codes are captured for each hospital stay using the Swiss classification of surgical interventions (CHOP). The full CHOP code is available online: http://tinyurl.com/mwzux9xb (accessed on 13 April 2024). The main CHOP code for endovascular implantation of a stent graft in the iliac artery, “39.79.12”, was only available since 2011. Before 2011, only unspecific coding for endovascular therapy without anatomical location was available. In addition, there are codes for endovascular coil embolization or occlusion of abdominal vessels “39.79.26” and for other extracranial vessels (“x.20 and x.29”) available. Since 2014, the additional codes “39.78.11, x.12, x.13, x.19” were added to differentiate between iliac stent grafts without a side branch (“x.11”), iliac stent grafts with a side branch (“x.12”), iliac stent graft with fenestration (“x.13”), and other stent graft (“x.19”). The CHOP codes for open surgical treatment were “38.36.17”, “39.25.11, x.12, x.19, x.21, x.22, x.99”, and “39.57.48”. These codes did not change during the observed period. Further details on ICD and CHOP codes are available in the Supplementary Material.

Data are fully anonymized due to personal data protection regulations. Thereby, each patient receives a new unique identifier for each admission. This obscures the identification of readmissions of the same patient. Further, the institution number is encoded and grouped into five levels of care, with level one indicating university hospitals, level two indicating larger non-university hospitals (“major hospitals”), and levels three to five indicating smaller hospitals for secondary care (“regional hospitals”) [12]. The analysis of this fully anonymized dataset did not require ethical approval (waived by the local ethics board: BASEC-Nr. Req-2021-01010). This study is reported in accordance with the STROBE statement [13].

### 2.2. Inclusion and Exclusion Criteria

All cases with “I72.3” as a primary or secondary diagnosis (ICD-10: aneurysm or dissection of the iliac artery; ADIA) were identified during the reporting years 2011–2018. Several additional filters were applied to identify cases that were truly admitted for this diagnosis. The Supplementary Table S1 provided an overview on the patient identification process. In short, cases with ADIA as a secondary diagnosis only and without a CHOP code for surgical treatment were excluded. Likewise, all elective admissions with ADIA as primary diagnosis but without a CHOP code for surgical treatment were excluded. Further, all cases with a primary diagnosis of any aortic aneurysm (ICD I71.3–I71.6) or any aortic dissection (ICD I71.00–I70.07), as well as all cases with both endovascular and open surgical treatment codes were excluded. Finally, all acute admissions without a surgical treatment code and with discharge to another acute care hospital were excluded to avoid duplicates. 

### 2.3. Statistical Analysis

Factor variables were summarized with counts and percentages and compared using the Chi-squared test. Continuous variables were summarized with the median and quartiles 25 and 75 and compared using the Kruskal–Wallis rank test. Comorbidities were summarized using a sum score of weighted Elixhauser ICD-10 diagnosis groups according to van Walraven [14]. This involves evaluating the presence of ICD-10 codes within each comorbidity category for every case, and then aggregating them using a weighting system using the “comorbidity” R package version 0.5.3 by Gasparini [15]. The used ICD-10 codes are available in the Supplementary Tables S2 and S3. Hospital incidence rates were age-standardized using the 2013 European standard population and for the Swiss population data from the SFSO as previously described [16,17,18]. 

A multivariable logistic regression model was built to analyze the association between treatment modality and hospital mortality. The continuous variables of age and the van Walraven comorbidity score, as well as the factor variables of sex, type of admission, type of treatment, insurance class, hospital level, and period of treatment (2011–2014 vs. 2015–2018) were included in this model to adjust for potential confounding. Cases without surgical treatment were excluded. Regression coefficients were presented using odds ratios (OR) and the corresponding 95% confidence intervals (95%-CI). Hospital levels from 3 to 5 were merged to obtain a reasonable number of patients in this group. The data structure does not allow for missing data. All analyses were performed using R version 4.2.3 on macOS 12.5.1 [19]. All *p*-values were two-sided with an alpha-level of 5%. 

## 3. Results

**Figure 1:**
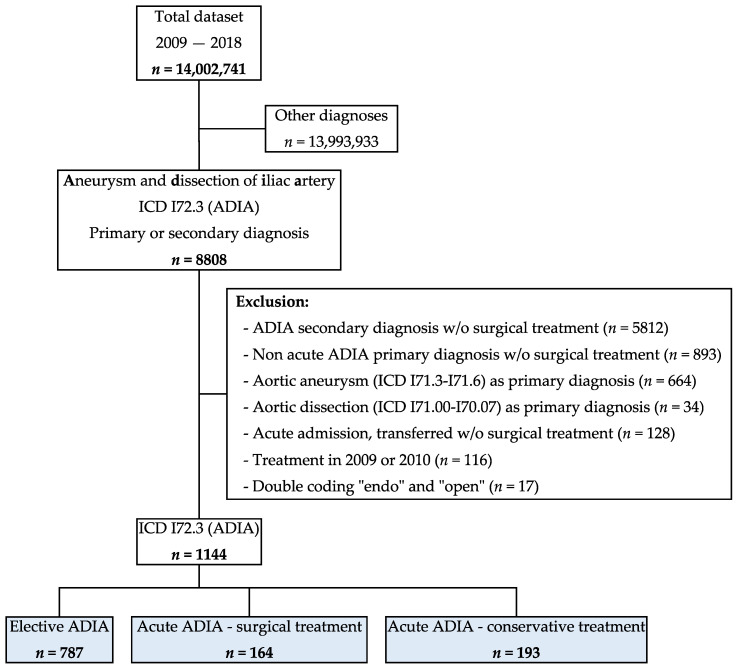
The total dataset contained all hospitalizations in the Swiss population in the years from 2009 to 2018. ICD = International Classification of Diseases (version 10); ADIA = aneurysm and dissection of iliac artery. w/o = without.

From 01.01.2011 to 31.12.2018, 8808 cases were hospitalized with ADIA as a primary or secondary diagnosis. After excluding 7664 cases, a total of 1144 cases were included in this study. Figure 1 details the patient flow with reasons for exclusion. In total, 787 (68.8%) were electively treated for ADIA, 164 (14.3%) received urgent or emergent treatment for ADIA, and the remaining 193 (16.9%) received conservative treatment for ADIA. Data were complete for the published variables.

Table 1 and Table 2 show the baseline characteristics of electively treated cases and surgically treated emergencies, each stratified by treatment modality. Cases receiving endovascular therapy were significantly older compared to cases that received open surgical repair: 74 versus 69 years in the elective cases, *p* < 0.001, and 75 versus 72.5 years in emergency cases, *p* = 0.02. In the elective setting, the proportion of endovascular treatment was significantly higher in the later treatment period (2015 to 2018) compared to the more historic period (2011 to 2014), *p* < 0.001. The same tendency was seen in the emergency setting but did not reach statistical significance. The change in treatment modality is illustrated by the Supplementary Figure S1. The overall proportion of endovascular repair steadily increased from 20.4% in 2011 to 58.7% in 2018, whereas the proportion of open repair (46.6% to 24.5%) and conservative management (33.0% to 16.8%) decreased during the same period. Characteristics and hospital mortality of the acute cases with ADIA without surgical therapy are summarized in the Supplementary Table S4.

### 3.1. Epidemiology

**Figure 2:**
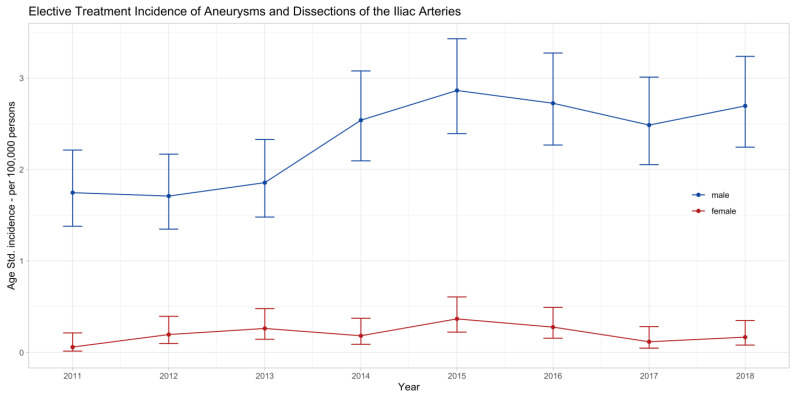
Age-standardized incidence rates of elective treatment of iliac artery aneurysms or dissections in Switzerland between 2011 and 2018 with 95% confidence intervals stratified by sex. The incidence significantly increased in males (*p* = 0.012) and was stable in females (*p* = 0.674) in the observed eight years.

The age-standardized incidence rates for elective surgical treatment of ADIA in Switzerland are plotted in Figure 2. The incidence rates were about 10 times higher in men than in women. They significantly increased in men from 1.75 (95%-CI: 1.4 to 2.2) to 2.7 (2.2 to 3.2) cases per 100,000 men, *p* = 0.012, and were stable in women at around 0.2 (0.1 to 0.4) per 100,000 women, *p* = 0.674. 

**Figure 3:**
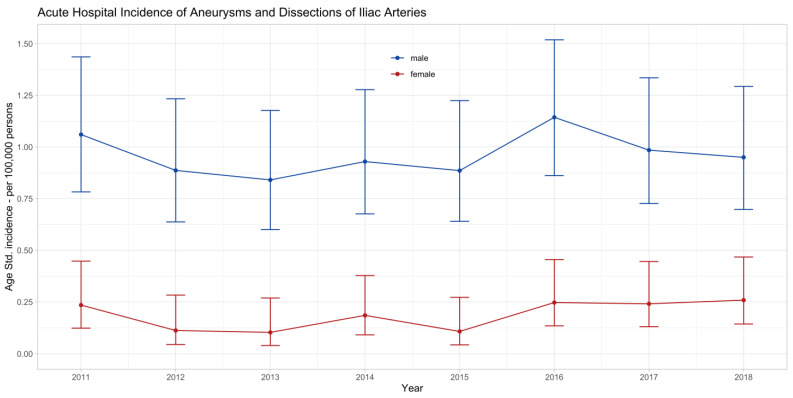
Age-standardized incidence rates of all emergent admissions for iliac artery aneurysms or dissections in Switzerland between 2011 and 2018 with 95% confidence intervals stratified by sex (both surgically treated and conservatively managed). The incidence rates were stable in the observed eight years in both males (*p* = 0.688) and females (*p* = 0.212).

The age-standardized incidence rates for emergent hospital admission for ADIA in Switzerland are plotted for 2011 to 2018 in Figure 3. These figures included both surgically treated cases and cases with conservative management. The incidence rates were about five times higher in men than in women. They were stable in both sexes in the observed period at around 0.9 (95%-CI: 0.7 to 1.3) cases per 100,000 men and around 0.2 (0.1 to 0.4) cases per 100,000 women. 

### 3.2. Treatment Modality

**Figure 4:**
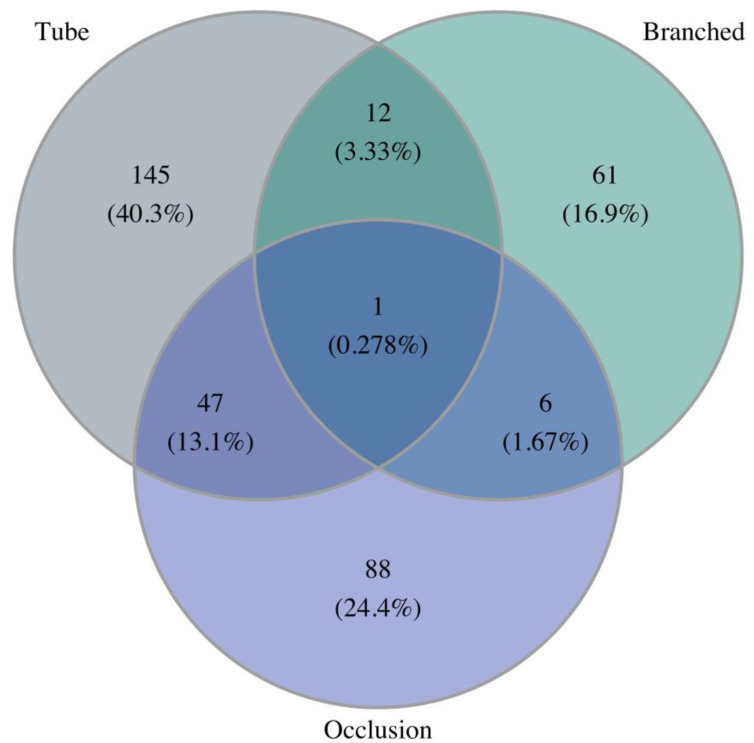
Venn diagram showing coding of endovascular therapy for elective or acute treatment of ADIA for the years from 2014 to 2018, excluding 3 cases coded with a fenestrated graft.

There was a steady increase in endovascular therapy in the observed period. In 2011, only 27% of the cases were treated using an endovascular approach, where it was 63.3% in 2018. On the other hand, the proportion of conservatively managed cases and cases managed with open surgical treatment decreased in the period from 29.7% to 14.2%, and from 43.2 to 22.5%, respectively. A figure detailing these proportions is available in the Supplementary Material. Figure 4 shows an overall Venn diagram of the type of endovascular therapy including elective and emergency treatments. Subcodes for tube, branched, or fenestrated grafts were only available for the years from 2014 to 2018. Three cases were coded with a fenestrated graft, and these were excluded to increase readability. Most cases were coded with tube stent graft implantation (40.3%), the second largest group was coded with an isolated vessel occlusion (24.4%), and an additional 16.9% were coded to have a branched device implanted without additional vessel occlusion. A total of 47 cases (13.1%) were coded with endovascular occlusion and tube graft implantation. All other combinations were relatively seldom.

### 3.3. Treatment Outcomes—Elective

Table 3 summarizes the procedural details and treatment outcomes of cases with elective treatment for ADIA. There were significant differences in both the length of intensive care unit admission and length of hospital stay in favor of endovascular therapy, both *p* < 0.001. Likewise, the proportion of cases needing packed red blood cell transfusion was significantly higher after open surgical repair than endovascular repair, *p* < 0.001. In general, the number of reported complications was low for both endovascular and open repair, except for lower limb ischemia, with 6.5% of cases after endovascular repair and 14.2% after open repair, *p* < 0.001. Despite the high rate of lower limb ischemia, crural fasciotomy was only coded in 0.6%, and major amputation was never coded. 

### 3.4. Treatment Outcomes—Emergency 

Table 4 summarizes the procedural details and treatment outcomes of cases with emergency surgical treatment for ADIA. Like the elective setting, the length of intensive care stay, and the total length of hospital stay were significantly lower after endovascular repair. On the other hand, the need for transfusion was similar in both groups. Complication rates were dramatically higher than in the elective setting, with an overall rate of myocardial infarction of 4.9% compared to 0.4% in the elective setting. Notably, mesenteric ischemia was coded significantly more often after open surgical repair than after endovascular therapy, 12% versus 2.2%, *p* = 0.012. Likewise, small- and large-bowl resection was coded more often after open surgery than after endovascular treatment, without reaching statistical significance. Lower limb ischemia and major amputation were quite common and seen after both endovascular- and open-surgical repair without statistically significant differences.

### 3.5. Hospital Mortality

The crude hospital mortality rates for elective treatment of ADIA were 0.8% after endovascular repair and 3.1% after open surgical repair, *p* = 0.022. For surgical emergency treatment, the crude hospital mortality rates were 9.9% after endovascular repair and 19% after open repair, *p* = 0.089. The hospital mortality rate in the conservatively treated cohort was 27%; see Supplementary Table S4.

**Figure 5:**
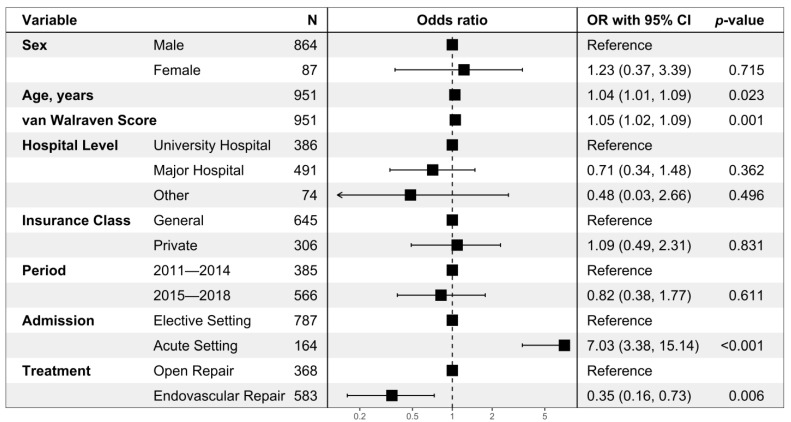
Multivariable logistic regression model on hospital survival. Data are complete. Analysis of 951 cases with 36 events (=hospital deaths). OR = odds ratio; 95% CI = 95% confidence interval of odds ratio.

The differences in treatment outcomes between open and endovascular repair cases were analyzed in a multivariable logistic regression analysis; see Figure 5. Endovascular repair was associated with a significantly reduced hospital mortality compared to open repair, OR 0.35 (0.16 to 0.73, *p* = 0.006). The adjusted mortality rate was significantly higher in the acute setting compared to elective treatment, OR 7.03 (3.38 to 15.14, *p* < 0.001). Further, increasing age at the time of treatment, OR 1.04 (1.01 to 1.09) per year, *p* = 0.023, and an increasing van Walraven score, OR 1.05 (1.02 to 1.09) per point, *p* = 0.001, were associated with higher hospital mortality. Sex, hospital level, insurance class, and period of treatment were not significantly associated with hospital mortality. 

## 4. Discussion

This is the first study to show comprehensive nationwide epidemiological data on the surgical treatment of iliac artery pathologies. The study shows several remarkable findings. First, the treatment incidence rate for men was roughly 10 times higher in the elective setting compared to women but only roughly five times higher for emergency treatments. Second, the data show favorable hospital outcomes for endovascular procedures compared to open surgical treatments. And third, there was no statistically significant difference in hospital treatment outcomes between men and women. 

An important note is required when interpreting these findings; in contrast to aortic pathologies, the current ICD coding for diseases of the iliac arteries does not differentiate between dissections and aneurysms. Therefore, there is also no classification between asymptomatic, symptomatic, or ruptured aneurysms of the iliac artery. The grouping into elective and acute cases was determined in this study by coding the hospitalization as either elective or emergency. An indirect verification of this grouping by the coding of hemorrhagic or hypovolemic shock (ICD R57.1) did not provide any additional information, as it is not clear whether the condition was present on admission or occurred during treatment as a complication of an originally elective treatment. Therefore, the specific mortality rate for the conservative treatment of iliac artery dissections as well as the specific mortality rate for symptomatic or ruptured iliac artery aneurysms remain unknown as these pathologies cannot be distinguished. The comparatively low hospital mortality in the “conservative management cohort” for acute ADIA of only 27% indicates that there might be a substantial proportion of dissections captured that were treated with the best medical therapy only. This hypothesis is also supported by the fact that another investigation for ruptured AAA (rAAA) has shown a hospital mortality rate in the palliative cohort of 95.7%. It seems very likely that the hospital mortality rate for palliative cases in patients with ruptured IAA might be at a comparable level [20]. 

### 4.1. Epidemiology

There is currently no comprehensive epidemiological data available to put our findings in an international context. The incidence of ADIA was roughly 10% of the incidence rates for AAA in the same period in Switzerland (2009–2018 for AAA versus 2011–2018 for ADIA) [16]. Comparison with historic data from a single institution in Switzerland (1972–1988) reported 7% of isolated IAA within all aorto-iliac aneurysms [21]. Both rates are substantially higher than the historic Swedish autopsy data that observed a prevalence of isolated IAA of only 0.7% of all aorto-iliac aneurysms [2]. The comparison is however difficult since this study describes treatment incidence, whereas the Swedish data described aneurysm prevalence in autopsies with a definition of ≥1.5 cm diameter. 

While the treatment incidence rates for AAA were stable in the last decade in Switzerland, there was a significant increase in elective treatment incidence in men for ADIA. On the other hand, the incidence rates for acute ADIA remained stable in both sexes. A possible and very likely explanation for the significant increase in the frequency of treatment in men is the increasing use of less invasive endovascular therapies, which led to the rise in the number of patients eligible for elective IAA repairs [22,23]. This is emphasized by the fact that the endovascularly treated cases were six years older than open repair cases. However, this argument would also apply to women, but the absolute number of cases may need to be bigger to capture any trends in females.

Another remarkable finding is that the proportion of females was substantially higher in emergency cases (15%) compared to elective cases (8%). A possible explanation could be a higher proportion of dissections in the emergency cohort, in which the proportion of women could also be higher. It is well-established for aortic diseases that, the proportion of females is higher in dissections compared to aneurysms [16,24]. 

The epidemiological data also show a decrease in the proportion of conservatively managed patients (Supplementary Figure S1). Reasons for this finding might be an increase in surgical treatment for dissections of the iliac arteries. Advances in diagnostics allow for better visualization of dynamic obstructions due to intimal flaps that might cause intermittent claudication [10]. On the other hand, there is emerging evidence that the risk for rupture of IAA might be lower than previously assumed [6,7]. Therefore, the recently published ESVS guidelines on this subject increased the diameter indication threshold from 3.5 cm to 4.0 cm [1]. It will be interesting to see whether these changes also have an impact on the number of ruptures or only reduce the number of elective procedures performed. Again, comprehensive clinical data from registries rather than administrative data are needed to answer this question.

### 4.2. Treatment Outcomes

The multivariable-adjusted analysis showed an approximate three times lower hospital mortality after endovascular repair than open surgical repair (OR 0.35, 95% CI: 0.16 to 0.73, *p* = 0.006). On the other hand, emergency procedures were associated with a seven times higher hospital mortality rate (OR 7.03, 95%-CI: 3.38 to 15.14, *p* < 0.001). This is not surprising, as higher hospital mortality rates for ruptured IAA must be expected. In line with previously published analyses on DRG data for different aortic pathologies in Switzerland, higher age, and an increase in the van Walraven score were significantly associated with hospital mortality [16,20]. In contrast to these earlier analyses, however, there were no differences between men and women in treating ADIA in Switzerland.

Interestingly, we observed an unexpectedly high rate of mesenteric complications following elective open surgery for ADIA. Acute mesenteric ischemia was diagnosed in 2.7% of cases undergoing open repair, compared to only 0.6% following endovascular therapy (*p* = 0.024). Large bowel resection was performed in 2.4% of cases after open repair, as opposed to only 0.2% after endovascular repair (*p* = 0.005). Additionally, small intestine resection occurred in 1% of cases after open repair, while it was only 0.4% after endovascular repair (*p* = 0.369). These complications are all serious; unfortunately, the dataset does not allow us to definitively establish their etiology. Mesenteric ischemia following treatment of the iliac arteries is presumably most often related to an insufficient collateral network in situations where the IIA is occluded intentionally or as a bailout in complicated situations [11]. The importance of IIA patency is underlined by the clear recommendation (Class I, Level C) that blood flow to at least one IIA should be preserved during either open or endovascular repair of IAA [1]. Besides the potential risk for colonic ischemia, other complications associated with IIA occlusion are buttock claudication, erectile dysfunction, and spinal cord ischemia [25]. Knowledge on an impaired collateral network due to occlusion of the contralateral IAA or the inferior mesenteric artery is essential when planning ADIA treatment but also when comparing treatment outcomes. This information is not available in our data and thus hinders us from drawing any conclusions on the risk of a specific treatment with these dramatic complications. Of note, acute mesenteric ischemia was coded in one of the 178 cases (0.6%) treated with coiling and in another two of the 314 cases (0.6%) that had endovascular therapy coded without coiling. Alternative explanations for mesenteric ischemia may also be intestinal hypoperfusion due to reduced perioperative arterial blood flow, especially in cases of hemorrhagic shock, so-called non-occlusive mesenteric ischemia (NOMI) [26]. Furthermore, concomitant acute mesenteric ischemia can also occur unrelated to the procedure itself during the hospital stay in patients with arteriosclerotic disease [27].

Within its limitations, this study provides a comprehensive picture of the epidemiology and periprocedural outcomes of patients treated for iliac artery pathology. As with many less invasive endovascular treatment alternatives, this study shows lower hospital mortality. However, this study lacks important information on the follow-up of these patients. Endovascular treatment of isolated IAA has been associated with higher rates of re-intervention than open surgical repair in cohort studies [28]. Further studies should focus on long-term outcomes and evaluate the burden of possible re-interventions and late complications to obtain a complete picture of the treatment for iliac artery pathologies. An individualized treatment decision must consider patient characteristics including anatomical parameters and patient preferences. In this shared decision, all perioperative benefits must be weighed against a higher rate of potential re-interventions.

### 4.3. Limitations

There are several limitations to this analysis. First, the reported cohort is heterogeneous and includes both aneurysms and dissections. The ICD coding does not allow for the differentiation between aneurysms and dissections. Further, there is no specific ICD code for ruptured IAA. Identification of the cases with acute pathologies was indirectly achieved via a variable for admission. Thus, treatment-specific outcomes are reported rather than disease-specific outcomes.

Secondly, these administrative data do not include the cardiovascular risk profile, the functional capacity of the treated individuals, the hemodynamic situation in dissections or ruptures, or anatomical characteristics of the pathology. Hence, adjustments were only possible for age, sex, the van Walraven comorbidity score, and the insurance state. Such unmeasured factors could explain the observed differences in survival rates to some extent and could theoretically explain the differences. 

Thirdly, the data allow non-independent observations of patients treated twice for the same ICD code. It must be assumed that some individuals were treated for both sides at different hospital admissions. The data structure does not allow for the identification of these cases; thus, it is likely that some non-independent observations are present in this cohort. 

Fourthly, we present epidemiological data on hospital admissions and treatments in Switzerland. Incidence rates depend not only on the disease itself, but also on less generalizable factors such as the organization and reimbursement of costs in the Swiss healthcare system.

Lastly, coding errors cannot be excluded, and the anonymized data structure does not allow for data validation. Nevertheless, the data provide almost complete coverage of the Swiss population since reimbursement depends on reporting the cases to the Swiss authorities. Selection bias and the risk of information bias in hard outcomes such as hospital mortality is therefore low [29]. 

## 5. Conclusions

This nationwide study of iliac artery pathologies shows that the treatment incidence was about 10 times higher in men than in women for elective procedures, but only about five times higher for emergency treatment. Hospital mortality rates were dramatically higher in emergency procedures compared to elective procedures. Endovascular procedures were associated with significantly lower hospital mortality than open procedures, while hospital mortality rates were comparable for men and women.

## Figures and Tables

**Table 1:** Baseline characteristics of elective cases.

Variable	Endovascular Repair (*n* = 492)	Open Repair (*n* = 295)	Total (*n* = 787)	*p* Value
Male sex	455 (92)	269 (91)	724 (92)	0.518
Age, years	75 (68, 80)	69 (63, 75)	73 (66, 79)	<0.001
van Walraven score	2 (0, 6)	3 (0.5, 10)	2 (0, 8)	<0.001
Coronary artery disease	114 (23)	78 (26)	192 (24)	0.301
Chronic heart failure	19 (3.9)	15 (5.1)	34 (4.3)	0.414
Cerebrovascular disease	30 (6.1)	18 (6.1)	48 (6.1)	0.998
Arterial hypertension	246 (50)	144 (49)	390 (50)	0.747
Chronic pulmonary disease	41 (8.3)	29 (9.8)	70 (8.9)	0.475
Diabetes mellitus	61 (12)	32 (11)	93 (12)	0.514
Chronic kidney disease	85 (17)	50 (17)	135 (17)	0.906
Cancer	7 (1.4)	7 (2.4)	14 (1.8)	0.329
Obesity	16 (3.3)	8 (2.7)	24 (3.0)	0.670
Type of hospital				0.045
University hospital (Level 1)	181 (37)	135 (45.8)	316 (40)	
Major hospital (Level 2)	267 (54)	138 (46.8)	405 (51)	
Other (Level 3 to 5)	44 (8.9)	22 (7.5)	66 (8.4)	
Location before admission				0.182
Home	455 (92)	272 (92)	727 (92)	
Acute care hospital	34 (6.9)	19 (6.4)	53 (6.7)	
Nursing home	2 (0.4)	0 (0.0)	2 (0.3)	
Other	1 (0.2)	4 (1.4)	5 (0.6)	
Treatment period				<0.001
2011–2014	115 (29.3)	162 (54.9)	277 (40.3)	
2015–2018	278 (70.7)	133 (45.1)	411 (59.7)	

Data are complete. Counts are presented with percentages and compared using Chi2 tests. Continuous variables are summarized with median and percentiles 25 and 75 and compared using Kruskal–Wallis rank tests. ICD-10 codes to identify comorbidities are available in the supplement.

**Table 2:** Baseline characteristics of surgically treated emergent cases.

Variable	Endovascular Repair (*n* = 91)	Open Repair (*n* = 73)	Total (*n* = 164)	*p* Value
Male sex	74 (81)	66 (90)	140 (85)	0.102
Age, years	74 (65, 81)	73 (60, 78)	73 (62, 80)	0.154
van Walraven score	10 (2, 19)	10 (3, 17)	10 (3, 18)	0.984
Coronary artery disease	22 (24)	17 (23)	39 (24)	0.894
Chronic heart failure	10 (11)	6 (8.2)	16 (9.8)	0.552
Cerebrovascular disease	5 (5.5)	1 (1.4)	6 (3.7)	0.227
Arterial hypertension	35 (38)	27 (37)	62 (38)	0.846
Chronic pulmonary disease	14 (15)	12 (16)	26 (16)	0.854
Diabetes mellitus	7 (7.7)	9 (12)	16 (9.8)	0.320
Chronic kidney disease	26 (29)	20 (27)	46 (28)	0.868
Cancer	3 (3.3)	0 (0)	3 (1.8)	0.254
Obesity	1 (1.1)	1 (1.4)	2 (1.2)	0.999
Type of hospital				0.668
University hospital (Level 1)	42 (46)	28 (38)	70 (43)	
Major hospital (Level 2)	45 (49)	41 (56)	86 (52)	
Other (Level 3 to 5)	4 (4.4)	4 (5.5)	8 (4.9)	
Location before admission				0.196
Home	69 (76)	59 (81)	128 (78)	
Acute care hospital	16 (18)	14 (19)	30 (18)	
Nursing home	2 (2.2)	0 (0)	2 (1.2)	
Other	4 (4.4)	0 (0)	4 (2.4)	
Treatment period				0.487
2011–2014	35 (38)	32 (44)	67 (41)	
2015–2018	56 (62)	41 (56)	97 (59)	

Data are complete. Counts are presented with percentages and compared using Chi2 tests. Continuous variables are summarized with median and percentiles 25 and 75 and compared using Kruskal–Wallis rank tests. ICD-10 codes to identify comorbidities are available in the supplement.

**Table 3:** Treatment details and outcomes of elective cases.

Variable	Endovascular Repair (*n* = 492)	Open Repair (*n* = 295)	Total (*n* = 787)	*p* Value
Length of stay ICU, hours	0 (0, 0)	16 (0, 26)	0 (0, 19)	<0.001
Length of hospital stay, days	4 (3, 6)	9 (7, 14)	6 (3, 10)	<0.001
Packed red blood cells				<0.001
0	430 (87)	201 (68.1)	631 (80)	
1–5	46 (9.3)	66 (22.4)	112 (14)	
>5	16 (3.3)	28 (9.5)	44 (5.6)	
Fresh frozen plasma				0.010
0	487 (99)	283 (95.9)	770 (98)	
1–5	4 (0.8)	10 (3.4)	14 (1.8)	
>5	1 (0.2)	2 (0.7)	3 (0.4)	
Platelet transfusion				0.609
0	491 (100)	294 (99.7)	785 (100)	
1–5	1 (0.2)	0 (0.0)	1 (0.1)	
>5	0 (0)	1 (0.3)	1 (0.1)	
Myocardial infarction	2 (0.4)	1 (0.3)	3 (0.4)	0.999
Acute mesenteric ischemia	3 (0.6)	8 (2.7)	11 (1.4)	0.024
Large intestine resection	1 (0.2)	7 (2.4)	8 (1.0)	0.005
Small intestine resection	2 (0.4)	3 (1.0)	5 (0.6)	0.369
Acute lower limb ischemia	32 (6.5)	42 (14.2)	74 (9.4)	<0.001
Crural fasciotomy	1 (0.2)	4 (1.4)	5 (0.6)	0.068
Major amputation	0 (0)	0 (0.0)	0 (0)	NA
Destination after discharge				<0.001
Home	443 (90)	240 (81.4)	683 (87)	
Rehabilitation	21 (4.3)	37 (12.5)	58 (7.4)	
Acute care hospital	14 (2.8)	8 (2.7)	22 (2.8)	
Nursing home	8 (1.6)	0 (0.0)	8 (1.0)	
Other	2 (0.4)	1 (0.3)	3 (0.4)	
Hospital mortality	4 (0.8)	9 (3.1)	13 (1.7)	0.022

Data are complete. Counts are presented with percentages and compared using Chi2 tests. Continuous variables are summarized with median and percentiles 25 and 75 and compared using Kruskal–Wallis rank tests. ICD-10 codes to identify comorbidities are available in the supplement. ICU = intensive care unit. Destination after discharge also includes mortality; this level is not shown as it is redundant with the hospital mortality variable. NA = not applicable (no events in both groups).

**Table 4:** Treatment details and outcomes for surgically treated emergent cases.

Variable	Endovascular Repair (*n* = 91)	Open Repair (*n* = 73)	Total (*n* = 164)	*p* Value
Length of stay ICU, hours	0 (0, 43)	46 (15, 109)	23 (0, 83)	<0.001
Length of hospital stay, days	11 (6, 19)	16 (10, 28)	13 (7, 21)	0.003
Packed red blood cells				0.171
0	43 (47)	26 (36)	69 (42)	
1–5	30 (33)	24 (33)	54 (33)	
>5	18 (20)	23 (32)	41 (25)	
Fresh frozen plasma				0.799
0	85 (93)	67 (92)	152 (93)	
1–5	4 (4.4)	5 (6.8)	9 (5.5)	
>5	2 (2.2)	1 (1.4)	3 (1.8)	
Platelet transfusion				0.254
0	88 (97)	73 (100)	161 (98)	
1–5	3 (3.3)	0 (0)	3 (1.8)	
>5	0 (0)	0 (0)	0 (0)	
Myocardial infarction	2 (2.2)	6 (8.2)	8 (4.9)	0.141
Acute mesenteric ischemia	2 (2.2)	9 (12)	11 (6.7)	0.012
Large intestine resection	2 (2.2)	6 (8.2)	8 (4.9)	0.141
Small intestine resection	1 (1.1)	4 (5.5)	5 (3.0)	0.173
Acute lower limb ischemia	9 (9.9)	14 (19)	23 (14)	0.089
Crural fasciotomy	2 (2.2)	4 (5.5)	6 (3.7)	0.408
Major amputation	2 (2.2)	2 (2.7)	4 (2.4)	>0.999
Destination after discharge				0.201
Home	54 (59)	37 (51)	91 (55)	
Rehabilitation	13 (14)	15 (21)	28 (17)	
Acute care hospital	11 (12)	4 (5.5)	15 (9.1)	
Nursing home	3 (3.3)	1 (1.4)	4 (2.4)	
Other	1 (1.1)	2 (2.7)	3 (1.8)	
Hospital mortality	9 (9.9)	14 (19)	23 (14)	0.089

Data are complete. Counts are presented with percentages and compared using Chi2 tests. Continuous variables are summarized with median and percentiles 25 and 75 and compared using Kruskal–Wallis rank tests. ICD-10 codes to identify comorbidities are available in the supplement. ICU = intensive care unit. Destination after discharge also includes mortality; this level is not shown as it is redundant with the hospital mortality variable.

## Data Availability

Data can be requested at the Swiss Federal Statistical Office: https://www.bfs.admin.ch/bfs/en/home/services/contact.html (accessed on 11 April 2024).

## Supplementary Materials

The following supporting information can be downloaded at: https://www.mdpi.com/article/10.3390/jcm13082267/s1, Supplementary Figure S1: Management of ADIA; Supplementary Table S1: Patient Identification; Supplementary Table S2: CHOP codes, Supplementary Table S3: ICD-codes for ComplicaTons; Supplementary Table S4: Characteristics and outcomes of conservative management for ADIA.
