# Supplementary material for: Hospital Incidence and Treatment Outcomes of Patients with Aneurysms and Dissections of the Iliac Artery in Switzerland—A Secondary Analysis of Swiss DRG Statistics Data
Source: J Clin Med. 2024 Apr 14;13(8):2267. doi: 10.3390/jcm13082267 (PMC11051054; doi:10.3390/jcm13082267)

Supplementary Table 1: Patient Identification

<table>
<tr><th></th><th colspan="2">Surgical Treatment (CHOP-Code)</th><th colspan="2">No Surgical Treatment (No CHOP-Code)</th></tr>
<tr><th></th><th>Acute Admission</th><th>Elective Admission</th><th>Acute Admission</th><th>Elective Admission</th></tr>
<tr><td>Primary Diagnosis ICD-I72.3</td><td>Include (n=111)</td><td>Include (n=653)</td><td>Include* (n=193)
• Excluded if discharged to another acute care hospital to avoid duplicates.

This heterogenous cohort includes palliative treatment of ruptured iliac aneurysms as well as conservative management for acute uncomplicated dissections of the iliac arteries.</td><td>Exclude (n=893)</td></tr>
<tr><td rowspan="2">Secondary Diagnosis ICD-I72.3</td><td>Include* (n=53)</td><td>Include* (n=134)</td><td colspan="2" rowspan="2">Exclude
(n= 5'812)</td></tr>
<tr><td colspan="2">• Only included if primary diagnosis was not aortic aneurysm (AAA = I71.3 or I71.4 or TAAA = I71.5 or I71.6)
• Only included if primary diagnosis was not aortic dissection (I71.00 - I71.07)</td></tr>
</table>

Supplementary Table 2: CHOP Codes

Code	**Open Repair** (translated, original available in German or French)
38.36.17	Resection of the iliac artery with anastomosis
39.25.11	Aorto-iliac bypass
39.25.12	Aorto-femoral bypass
39.25.19	Aorto-iliac-femoral Bypass, aortic
39.25.21	Iliac-iliac bypass
39.25.22	Iliac-femoral bypass
39.25.99	Aorto-iliac-femoral Bypass, other
39.57.48	Reconstruction of the iliac artery with synthetic implant
	**Endovascular Therapy** (translated, original available in German or French)
39.79.12	Endovascular implantation of stentgraft in iliac arteries
39.78.11	*Additional coding to 39.79.12, since 2014:* Tube graft without branch or fenestration
39.78.12	*Additional coding to 39.79.12, since 2014: Branched graft*
39.78.13	*Additional coding to 39.79.12, since 2014: Fenestrated graft*
39.78.19	*Additional coding to 39.79.12, since 2014: Other graft*
39.79.20	Coil-embolization or occlusion of vessel not further specified
39.79.26	Coil-embolization or occlusion of abdominal vessel
39.79.29	Coil-embolization or occlusion of vessel, other

Supplementary Table 3: ICD-Codes for Complications

**ICD-Code**	**Comorbidities**
DOI: 10.1097/MLR.0b013e31819432e5	Elixhauser Comorbidity Score (ES)
I25*	Chronic ischemic heart disease
I50*	Chronic heart failure
G45*, G46*, H340*, I6*	Cerebrovascular disease
Elixhauser Item No. 6	Hypertension
Elixhauser Item No. 9	Chronic pulmonary disease
Elixhauser Item No. 10 and 11	Diabetes mellitus
Elixhauser Item No. 13	Chronic kidney disease
Elixhauser Item No. 17, 18 and 19	Cancer
Elixhauser Item No. 22	Obesity

**ICD-Code**	**Complications**
I21.*, I22.*	Acute/Recurrent myocardial infarction
I74.*	Acute limb ischemia
K55.0	Acute mesenteric infarction

Supplementary Table 4: Conservative Management acute ADIA

Variable	Male (N=159)	Female (N=34)	Total (N=193)	p value
Age, years	77 (68, 84)	75 (68, 84)	77 (68, 84)	0.972
van Walraven score	5 (0, 14)	3 (0, 11)	5 (0, 13)	0.529
Coronary artery disease	33 (21)	8 (24)	41 (21)	0.72
Chronic heart failure	16 (10)	3 (8.8)	19 (9.8)	>0.999
Cerebrovascular disease	5 (3.1)	1 (2.9)	6 (3.1)	>0.999
Arterial hypertension	53 (33)	18 (53)	71 (37)	0.031
Chronic pulmonary disease	17 (11)	3 (8.8)	20 (10)	>0.999
Diabetes mellitus	12 (7.5)	1 (2.9)	13 (6.7)	0.471
Chronic kidney disease	40 (25)	5 (15)	45 (23)	0.191
Cancer	1 (0.6)	1 (2.9)	2 (1.0)	0.322
Obesity	4 (2.5)	0 (0)	4 (2.1)	>0.999
Type of hospital				0.842
University hospital	36 (23)	8 (24)	44 (23)	
Major hospital	98 (62)	22 (65)	120 (62)	
Other	25 (16)	4 (12)	29 (15)	
Location before admission				0.164
Home	133 (84)	26 (76)	159 (82)	
Nursing Home	1 (0.6)	2 (5.9)	3 (1.6)	
Other	9 (5.7)	2 (5.9)	11 (5.7)	
Acute care Hospital	16 (10)	4 (12)	20 (10)	
Treatment period				0.085
2011-2014	82 (52)	12 (35)	94 (49)	
2015-2018	77 (48)	22 (65)	99 (51)	
Length of hospital stay	8 (4, 15)	11 (3, 19)	9 (3, 16)	0.392
Mortality	46 (29)	7 (21)	53 (27)	0.323

Data were complete. Counts are presented with percentages and compared using Chi2-tests. Continuous variables are summarized with median and percentiles 25 and 75 and compared using Kruskal-Wallis rank tests. ICD-10 codes to identify comorbidities are available in the supplement. ICU = Intensive care unit. Destination after discharge also includes mortality; this level is not shown as it is redundant with the hospital mortality variable. NA = not applicable (no events in both groups).

Supplementary Figure 1: Management of ADIA

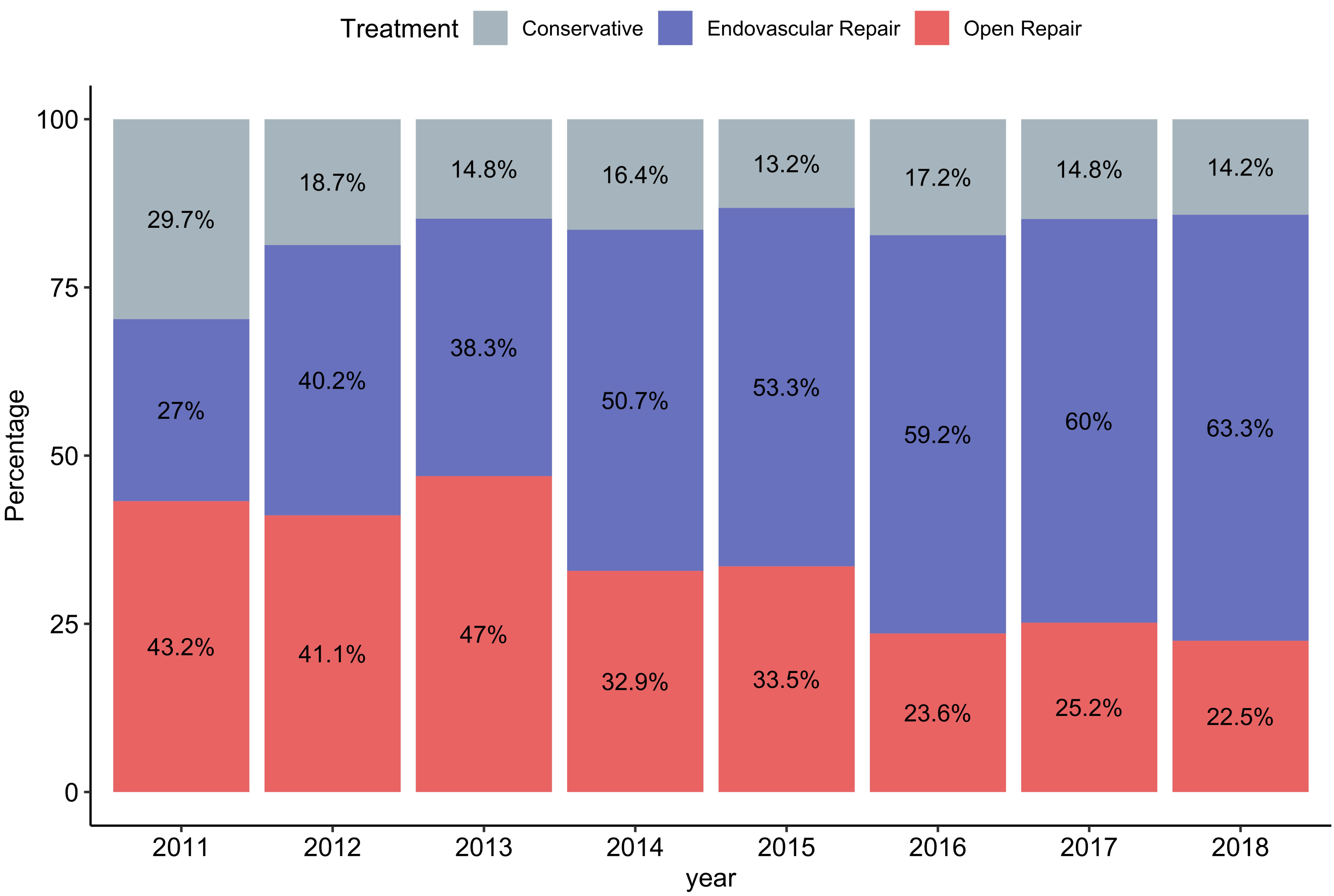

Supplement: Supplementary file 1 [file jcm-13-02267-s001.zip › jcm-2923213-supplementary.pdf]
